# Non-Invasive Retinal Pathology Assessment Using Haralick-Based Vascular Texture and Global Fundus Color Distribution Analysis

**DOI:** 10.3390/jimaging11090321

**Published:** 2025-09-19

**Authors:** Ouafa Sijilmassi

**Affiliations:** Optics Department, Faculty of Optics and Optometry, Complutense University of Madrid, C/Arcos de Jalón, 118, 28037 Madrid, Spain; o.sijilmassi@ucm.es

**Keywords:** fundus imaging, texture features, color histogram, vascular analysis

## Abstract

This study analyzes retinal fundus images to distinguish healthy retinas from those affected by diabetic retinopathy (DR) and glaucoma using a dual-framework approach: vascular texture analysis and global color distribution analysis. The texture-based approach involved segmenting the retinal vasculature and extracting eight Haralick texture features from the Gray-Level Co-occurrence Matrix. Significant differences in features such as energy, contrast, correlation, and entropy were found between healthy and pathological retinas. Pathological retinas exhibited lower textural complexity and higher uniformity, which correlates with vascular thinning and structural changes observed in DR and glaucoma. In parallel, the global color distribution of the full fundus area was analyzed without segmentation. RGB intensity histograms were calculated for each channel and averaged across groups. Statistical tests revealed significant differences, particularly in the green and blue channels. The Mahalanobis distance quantified the separability of the groups per channel. These results indicate that pathological changes in retinal tissue can also lead to detectable chromatic shifts in the fundus. The findings underscore the potential of both vascular texture and color features as non-invasive biomarkers for early retinal disease detection and classification.

## 1. Introduction

Fundus imaging is a specialized medical imaging technique used to capture detailed images of the retinal fundus, enabling comprehensive assessment of the retina, its vasculature, the optic nerve head, and the vitreous body. Leveraging mathematical models and advanced algorithms, digital image processing facilitates the measurement, analysis, and quantification of retinal abnormalities with high precision. This approach plays a pivotal role in the early detection and diagnosis of various ophthalmic diseases, notably glaucoma and diabetic retinopathy (DR) [1,2,3].

Glaucoma is a chronic, irreversible neurodegenerative disorder of the optic nerve, primarily characterized by the apoptotic loss of retinal ganglion cells and progressive axonal degeneration, ultimately resulting in vision loss. The disease also induces structural alterations in the retinal microvasculature, including decreased vascular density and narrowing of retinal vessel diameters [4]. On the other hand, DR, one of the most common microvascular complications of diabetes mellitus, remains the leading cause of blindness among working-age adults (20–65 years) [5]. In its early stages, DR is marked by the presence of retinal capillary microaneurysms, intraretinal microvascular abnormalities, and increased vascular permeability, often culminating in dot-blot hemorrhages [6]. Both glaucoma and DR may lead to vision impairment or irreversible blindness, frequently manifesting as structural abnormalities visible in fundus images. The retinal vasculature is crucial for delivering oxygen and nutrients to the retina and supporting the transmission of visual information from the retina to the brain [7]. Therefore, morphological changes in retinal vessels serve as critical biomarkers for the onset and progression of various ocular and systemic diseases, including glaucoma and DR.

In recent years, the availability of large volumes of retinal image data has increased significantly; however, much of the embedded clinical information remains underexploited. Texture analysis has emerged as a powerful tool for detecting and segmenting anatomical structures that exhibit subtle alterations in image texture, such as damage to the retinal vasculature. By quantifying the spatial distribution of intensity values within an image, texture analysis facilitates the comparison of similar regions across different images, offering critical insights into pathological changes in retinal morphology.

In the broader field of medical image analysis, texture analysis has demonstrated its utility in the evaluation of various imaging modalities, including Magnetic Resonance Imaging (MRI), Computed Tomography (CT), ultrasound, and X-ray, across multiple organ systems such as the heart, lungs, brain, kidneys, and liver [8,9,10]. Among texture analysis techniques, the method introduced by Haralick et al. [11] based on the Gray-Level Co-occurrence Matrix (GLCM), has gained widespread adoption, particularly in biomedical imaging. The GLCM captures second-order statistical information by quantifying the spatial relationships between pairs of pixels separated by a defined distance and orientation, thereby enabling the extraction of discriminative texture features.

In the present study, Haralick texture features were extracted and analyzed to identify the most salient attributes for detecting and differentiating vascular abnormalities associated with glaucoma and DR in comparison to normal retinal conditions. Retinal blood vessels were segmented from color fundus images using an automated extraction pipeline, enabling objective quantification of vascular anomalies across control and pathological groups. Furthermore, histogram analysis was applied to the full retinal background, capturing texture information based on color distribution across the RGB channels. This approach allows for a comprehensive evaluation of retinal background characteristics, offering additional context for identifying disease-specific patterns.

### Related Work

Recent advances in retinal image analysis have focused heavily on improving vascular segmentation and disease classification using both classical image processing and deep learning methods, which directly relate to the core components of this study. Traditional texture analysis remains a powerful tool for capturing spatial relationships in medical images. GLCM has been widely applied across various imaging modalities, including MRI, CT, ultrasound, and fundus imaging [8,9,12]. In retinal imaging, Haralick features have been explored for detecting vascular abnormalities; however, few studies have explicitly focused on segmented vessel structures for texture-based disease differentiation. The present research addresses this gap by concentrating on vessel-specific texture descriptors, enabling a more targeted and sensitive assessment of retinal pathology. Several prior works have leveraged classical image processing for retinal disease detection. Ahmad et al. [1] and Kumar et al. [2] explored statistical and morphological analysis for diabetic retinopathy and glaucoma detection. Color distribution has also been highlighted as a valuable diagnostic cue in studies by Stanley et al. [13] and Zhou et al. [14], which used RGB histograms for classification. Beyond texture and color, fractal geometry has emerged as a viable alternative for structural quantification. Igalla-El Youssfi and López-Alonso [15] proposed novel fractal and multifractal metrics for quantifying vascular complexity in diabetic retinopathy and glaucoma. Their results demonstrated that changes in multifractal spectra effectively capture disease progression, reinforcing the clinical utility of handcrafted and interpretable features especially those that describe topological or textural irregularities. Meanwhile, deep learning continues to push the boundaries of segmentation and classification. Karaali et al. [16] introduced DR-VNet, a Dense Residual U-Net tailored to thin and tiny vessels vessel segmentation. Wei et al. [17] proposed OCE-Net, integrating orientation-aware convolutions to maintain vascular continuity critical for downstream texture analysis. Li et al. [18] applied a multi-scale residual similarity gathering (MRSG) module to generate pixel-wise adaptive filters (PA-Filters), enhancing segmentation quality of retinal vessel images. On the classification front, Shamsan et al. [19] developed a hybrid classification method that integrates handcrafted features with deep learning representations to diagnose ocular diseases from color fundus imaging. This hybrid philosophy is echoed in the present study’s integration of vascular texture and color distribution features.

In summary, this work contributes to a body of literature that embraces both classical and modern techniques. By combining Haralick-based vascular texture with global RGB histogram analysis, a dual-feature, interpretable approach that aligns with recent trends while retaining computational simplicity and diagnostic transparency is presented.

## 2. Materials and Methods

### 2.1. Dataset

This study employed retinal fundus images from the publicly available High-Resolution Fundus (HRF) database [20], which provides high-quality color fundus images suitable for vascular and pathological analysis. The dataset comprises a total of 45 images, each with a resolution of 3504 × 2336 pixels, encoded in 24-bit color and stored in JPEG format with low compression to maintain high image quality.

**Figure 1:**
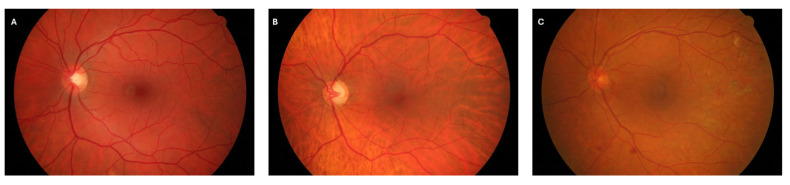
Example retinal fundus images from the High-Resolution Fundus (HRF) database [20], available under the Creative Commons 4.0 Attribution License (CC BY 4.0). (**A**) Healthy retina. (**B**) Retina with glaucoma. (**C**) Retina with diabetic retinopathy.

The HRF dataset is divided into three equal subsets (Figure 1):

- Healthy group: 15 images from subjects with no clinical signs of retinal disease.
- Diabetic Retinopathy (DR) group: 15 images exhibiting vascular abnormalities consistent with DR, such as microaneurysms and hemorrhages.
- Glaucoma group: 15 images from patients diagnosed with advanced-stage glaucoma, characterized by structural changes in the optic nerve head and retinal vasculature.

This dataset was selected due to its high resolution and well-annotated pathological categories, making it a robust resource for evaluating automated texture and vascular analysis techniques.

### 2.2. Features Extraction

Haralick texture features, derived from the Gray-Level Co-occurrence Matrix (GLCM), represent a well-established method for quantifying image texture. Their widespread use is attributed to both the computational simplicity of the method and the interpretability of the resulting descriptors. The concept was first introduced by Haralick et al. [21] who proposed a set of statistical features derived from the spatial relationships between pixel intensities within a grayscale image. Haralick’s approach is based on the premise that texture can be characterized by the frequency with which pairs of pixel values (grey levels) occur in specific spatial configurations.

The GLCM is constructed by computing how often a pixel with grey level *i* occurs in a defined spatial relationship (distance d and orientation angle *θ*) to a pixel with grey level *j*. Formally, the co-occurrence matrix element (*i*, *j*) reflects the number of times this pixel pair appears in the image under the specified conditions.

Given a grayscale image ***I*** of size $N_{x}\times N_{y}$, the co-occurrence value for grey levels *i* and *j* at a distance *d* and orientation *θ*, $P_{d,\theta }(i,j)$, can be defined as [22]:

(1) $$P_{d,\theta }(i,j)=\sum_{x=0}^{N_{x}-1}\sum_{y=0}^{N_{y}-1}\delta_{d,\theta ,i,j}(x,y)$$

where

(2) $$\delta_{d,\theta ,i,j}(x,y)=\{\begin{array}{cc} 1 & \begin{array}{cc} if & I(x,y)=i\begin{array}{cc} and & I(x+\pi_{x}(d,\theta ),y+\pi_{y}(d,\theta ))=j \end{array} \end{array}\\ 0 & Otherwise \end{array}$$

where I(x,y) represents the pixel value at position (*x*,*y*) in the quantized image. This matrix serves as the basis for extracting several second-order statistical features, such as contrast, correlation, energy, and homogeneity, that summarize texture characteristics across different regions of the image. These features are particularly relevant for characterizing pathological changes in retinal tissue, as they capture local structural variations that may not be apparent through intensity-based measures alone.

The offset (*π_x_*(*d*, *θ*), *π_y_*(*d*, *θ*)) defines the relative position of a pixel (*x*,*y*) with respect to its neighbor located at a distance *d* and orientation *θ*. In this study, the gray-level co-occurrence matrices (GLCMs) required for texture analysis were computed using MATLAB’s built-in graycomatrix function. Once the co-occurrence matrix is obtained, the corresponding Haralick texture features were extracted. The features considered in this work are those originally proposed by [21], as shown in Table 1.

Where $\mu_{x},\mu_{y}$ and $\sigma_{x},\sigma_{y}$ are the means and standard deviations, respectively, expressed as:

(11) $$\begin{array}{l} \mu_{x}=\sum_{i=1}^{N}i.P_{x}(i)\\ \mu_{y}=\sum_{j=1}^{N}j.P_{y}(j)\\ \sigma_{x}^{2}=\sum_{i=1}^{N}{\left(i-\mu_{x}\right)}^{2}P_{x}(i)\\ \sigma_{y}^{2}=\sum_{j=1}^{N}{\left(j-\mu_{y}\right)}^{2}P_{y}(j) \end{array}$$

In this study, second-order texture features derived from the GLCM, specifically Haralick features, were selected due to their ability to capture spatial dependencies and textural patterns within retinal images. Unlike first-order features, which rely solely on pixel intensity statistics without accounting for spatial relationships, second-order features analyze the joint probability of pairs of pixel intensities, offering a more comprehensive representation of vascular texture.

Although fractal and geometrical features can provide valuable information regarding structural complexity and vessel morphology, they often require highly precise segmentation and may be less sensitive to subtle textural variations in retinal fundus images. Therefore, the use of second-order features enables a more robust and discriminative quantification of vascular abnormalities associated with diseases such as diabetic retinopathy and glaucoma

To identify structural abnormalities in the retinal vasculature, the vascular tree was segmented from each fundus image using a MATLAB^®^-based function developed by Kumar R. [23]. This method combines matched filtering with an adaptive, iterative thresholding algorithm that analyzes the intensity histogram to separate vessel structures from the background. Specifically, the thresholding component calculates the mean intensities of the foreground and background classes iteratively, updating the threshold value until convergence is achieved. This approach improves robustness under variable lighting conditions and enhances vessel detection in cases of low contrast or faint vasculature.

To further improve segmentation performance, particularly in challenging images, all input images underwent preprocessing using contrast-limited adaptive histogram equalization (CLAHE) to enhance local contrast, and median filtering to suppress background noise while preserving edge integrity. These preprocessing steps enhanced vessel-background separability and facilitated more accurate vascular tree extraction.

All segmentation and subsequent texture analyses were performed using MATLAB^®^ software (version 2021b, The MathWorks Inc., Natick, MA, USA). Segmentation quality was assessed via visual comparison with the ground truth masks provided in the HRF database. Although quantitative validation metrics (e.g., Dice similarity coefficient) were not computed in this study, the visual inspections confirmed that the extracted vascular trees closely approximated the annotated references in most cases, supporting their suitability for downstream texture analysis.

Textural features were then extracted from the segmented vessels following the methodology proposed by [24], across eight Haralick texture metrics derived from the gray-level co-occurrence matrix (GLCM). Each image was first converted to 8-bit grayscale and quantized to 16 gray levels to reduce computational complexity while preserving texture patterns relevant for clinical analysis.

GLCMs were computed for a pixel distance (*d*) of 1 in four standard orientations: 0°, 45°, 90°, and 135°. The matrices were then normalized to represent co-occurrence probabilities (summing to 1), allowing for consistent comparison across images. To ensure directionally invariant feature extraction, Haralick features were calculated separately for each angle and then averaged across the four directions.

To statistically evaluate group differences between healthy and pathological retinas, the two-sample Kolmogorov–Smirnov test was applied using MATLAB’s built-in kstest2 function.

### 2.3. Histogram Analysis

A color image can be represented as a two-dimensional array of pixels, where each pixel consists of three components corresponding to the primary colors: Red (R), Green (G), and Blue (B). The RGB color space is widely used in digital imaging and represents an image as a numerical array of size *M* × *N* × 3, where *M* and *N* denote the height and width of the image, respectively, and the third dimension corresponds to the three-color channels. Each color channel is typically encoded using 8 bits per pixel, yielding intensity values in the range [0, 255], where 0 represents the absence of a given color and 255 corresponds to its maximum intensity [25]. The numerical range may vary depending on the data type used to store the image. A color histogram [26] characterizes the distribution of colors within an image by quantizing the color space and counting the frequency of each discrete color combination. This results in a probability distribution, where the *x*-axis represents the histogram bins (e.g., 0–255), and the *y*-axis indicates the number of pixels corresponding to each bin value. The number of bins is determined by the level of quantization and the number of distinct color values present. It is important to note that a histogram in the RGB color space is three times larger than one based solely on intensity values, due to the presence of three separate color channels. The histogram of an image, *I*(*x*,*y*), with height *ℎ* and width *w*, can be formally defined as follows [27,28]:

(12) $$n_{i}=\left|\left\{(x,y)|I(x,y)=i\right\}\right|,\;i=0,\ldots ,k-1$$

where *k* is the number of colors and $\sum_{i=0}^{k-1}n_{i}=w.h$.

All images used in this study were RGB color images with a resolution o9f 2336 × 3504 pixels and 8 bits per channel. To assess the normality of the data distribution, the lillietest function from MATLAB’s Statistics and Machine Learning Toolbox was employed. This function implements a variant of the Kolmogorov–Smirnov test, which evaluates whether a sample originates from a normally distributed population, based on the sample’s estimated mean and standard deviation. The results indicated that the data did not follow a normal distribution.

Subsequently, average histograms were computed by group and by color channel (R, G, B). The RGB histograms from the control and pathological groups were then compared using the non-parametric Wilcoxon-Mann-Whitney test, performed separately for each channel.

To further quantify differences between the two groups, the Mahalanobis distance was calculated between the average histograms of the normal and pathological images. This metric is particularly suitable for comparing multivariate distributions, as it accounts for correlations among variables and considers each group as a point cloud in a 256-dimensional feature space (one dimension per histogram bin).

Finally, to visualize the joint distribution of color intensities across channels, bivariate histograms were generated. These 2D histograms enable analysis of the co-distribution of two-color channels at a time, offering a more comprehensive view of RGB value relationships across the pixel data.

## 3. Results and Discussion

### 3.1. Texture Analysis

**Figure 2:**
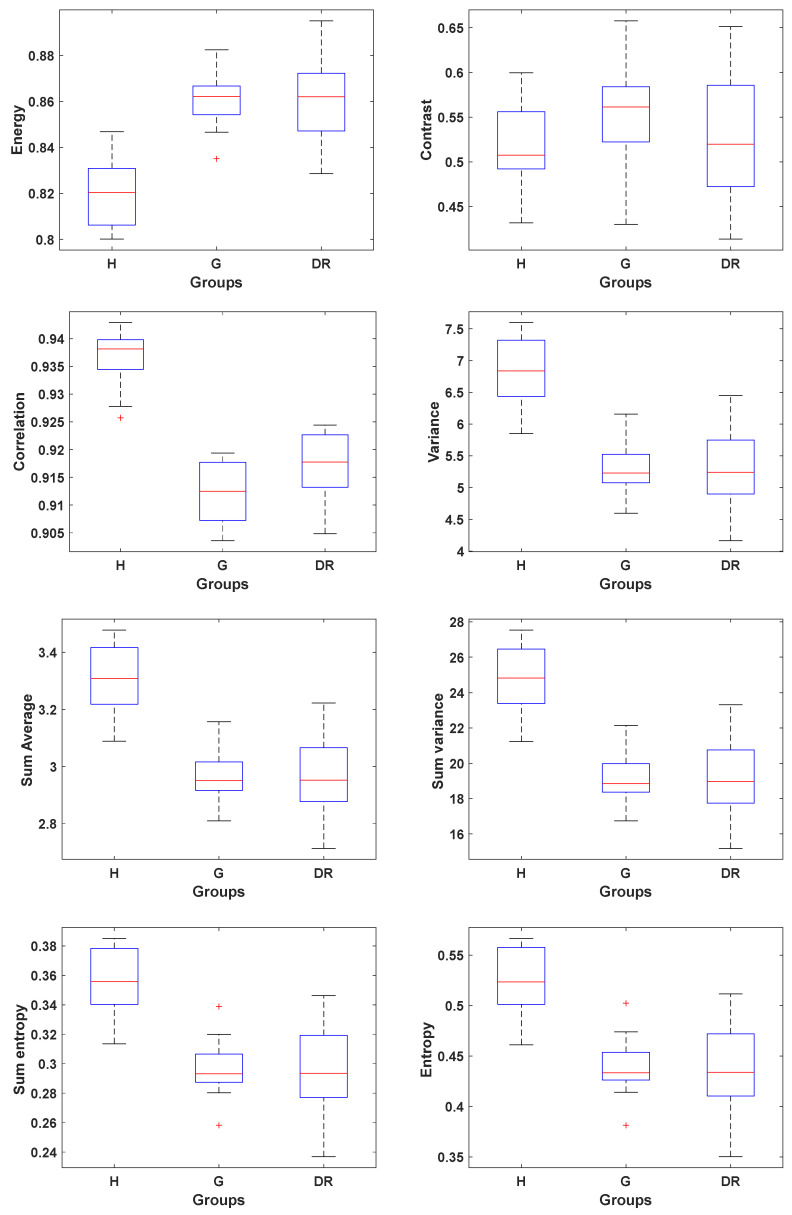
Box plot representation of the eight Haralick texture features extracted from the segmented vasculature, shown for healthy (H), glaucomatous (G), and diabetic (DR) groups. The ‘+’ symbol represents outliers, and the red line in each subfigure indicates the median.

Haralick texture features were calculated for each retinal image. A total of eight features were extracted from the Gray-Level Co-occurrence Matrix (GLCM): energy, contrast, correlation, variance, sum average, sum variance, sum entropy, and entropy. The mean and standard deviation (μ±σ) for each feature, along with the Kolmogorov–Smirnov test results comparing healthy individuals with patients diagnosed with glaucoma and DR, are presented in Table 2 and Figure 2, respectively.

Table 2 highlights the texture features that demonstrated statistically significant differences between healthy and pathological retinae vascularity. Specifically, the features correlation, variance, sum average, sum variance, sum entropy, and entropy were all significantly lower in both pathological groups compared to the healthy group. In contrast, energy was significantly higher in the glaucomatous and DR groups than in the control group.

Contrast quantifies local intensity variations within an image, ranging from 0 (indicating no contrast) up to a maximum value of ${\left(N_{g}-1\right)}^{2}$, where *N_g_* represents the number of gray levels determined by the image’s bit depth. This metric estimates the relative variation in luminance by correlating it with the intensity gradients present in the image. Higher contrast values correspond to greater variation among pixel intensities, reflecting more pronounced texture or edges [29]. Table 2 shows that all groups exhibited relatively similar values, suggesting general uniformity in vascular contrast across the sample.

Energy quantifies the uniformity or textural homogeneity of an image, with values ranging from 0 to 1. A value near 1 indicates a highly uniform image with minimal variation in pixel intensities, while a value close to 0 suggests high variability [30]. A high energy value implies that adjacent pixels have similar intensities, resulting in a smoother, less textured appearance [31]. In this study, pathological groups exhibited higher energy values compared to healthy retinae, indicating a more uniform distribution of grey levels. This feature reflects the degree of similarity between neighboring pixels. In the context of retinal vascularity, this suggests that the diseased retinae display more homogeneous textures or disorders in the texture, potentially due to reduced variation in vessel density or altered vascular organization. Such uniformity may be associated with structural changes in the retina. Glaucomatous eyes, for instance, are linked to a loss of structural complexity within the retinal vascularity [4]. Similarly, in DR, vessel branching tends to be reduced and vessels become narrower, leading to a more uniform and less complex vascular pattern [6]. The paper’s findings support the use of energy as a meaningful indicator of pathological changes in retinal vasculature.

Correlation measures the linear relationship between the intensity values of neighboring pixels in an image, ranging from −1 to 1, where 1 represents a perfect positive correlation, −1 a perfect negative correlation, and 0 no correlation. It provides insights into the regularity, alignment, and directionality of textures. A high correlation value indicates a strong linear dependency between adjacent pixel intensities, reflecting a more organized and coherent texture pattern. Conversely, a low correlation value suggests weaker pixel dependencies, indicating more disordered textures, suggesting disruptions in structural organization [31]. In the context of retinal imaging, correlation is closely linked to the spatial distribution and orientation of blood vessels. Structural disruptions caused by conditions such as DR or glaucoma can alter the regularity of vascular patterns, thereby reducing the correlation value. As such, changes in Haralick correlation serve as quantitative markers of retinal structural alterations, offering a way to numerically characterize the “smoothness” or “roughness” of the vasculature. This makes correlation a valuable feature for assessing disease-related changes in retinal architecture.

Variance is a statistical measure of texture heterogeneity that quantifies the dispersion of pixel intensity values within an image or region of interest. It ranges from 0 upwards, with higher values indicating greater variability in intensities, which reflects more complex and heterogeneous textures. Conversely, lower variance reflects more homogeneous textures, where pixel values are similar, a trait often associated with simpler or less structurally diverse tissues. In retinal imaging, reduced variance typically correlates with decreased vascular complexity. Conditions such as glaucoma and DR are characterized by diminished vascular density, reflected in fewer branches and capillary networks. This structural simplification leads to more uniform textures and therefore lower variance values. In DR, the observed decrease in vascular density is a manifestation of chronic microvascular damage, including capillary closure, dropout, and ischemia due to persistent hyperglycemia [32].

Entropy is a statistical metric that quantifies the degree of randomness, heterogeneity, and complexity in an image’s intensity distribution. It measures the unpredictability of pixel intensity values within a specified region, with values ranging from a minimum of 0 indicating complete uniformity to a maximum of $\log_{2}(N_{g})$. When entropy is high (close to the maximum $\log_{2}(N_{g})$), it indicates that the distribution of gray levels is more uniform, with many different intensities present in an unpredictable manner. This high variability leads to greater visual complexity and a sense of structural disorder or heterogeneity, reflecting more chaotic textures or less predictable patterns. Lower entropy values correspond to more homogeneous textures with less variation. In the context of retinal vasculature, decreased entropy suggests reduced structural complexity. Comparative analysis between healthy individuals and patients with retinal pathology, such as glaucoma or DR, reveals that pathological retinae tend to exhibit lower entropy values. This reduction is associated with a loss of vascular complexity, including decreased capillary density, thinner vessels, and vessel dropout or occlusion. These structural changes lead to more uniform textures and diminished randomness in vascular patterns. Previous studies have shown that entropy-based analysis can enhance the detection of pathological regions in fundus images, particularly in DR, by identifying subtle textural alterations associated with microvascular damage [33].

To ensure the reliability of the texture features extracted, particularly entropy and Haralick-based metrics, it was first necessary to validate the accuracy of the vessel segmentation. The Dice Similarity Coefficient (DSC) was used to evaluate the accuracy of retinal vessel segmentation by comparing the results with the ground truth provided in the dataset. The DSC is a widely used performance metric that quantifies the similarity between two regions, in this case, the segmented retinal vessels and the corresponding ground truth annotations. The coefficient ranges from 0 to 1, where values closer to 1 indicate greater overlap and, therefore, higher segmentation accuracy.

The proposed method achieved a mean DSC of 0.86 ± 0.01, demonstrating a high degree of consistency with the ground truth. These results confirm the effectiveness and reliability of the segmentation process, which serves as a critical step for the subsequent extraction of Haralick-based texture features.

In this research, Haralick texture features were employed to quantitatively analyze the retinal vasculature with the aim of distinguishing between healthy and pathological retinae. These features capture subtle variations in vascular texture, allowing for a robust differentiation of structural patterns associated with disease progression. The results demonstrate that Haralick texture analysis is both feasible and informative for identifying retinal abnormalities. However, no correction for multiple comparisons was applied, as the study was exploratory in nature and based on a limited dataset. Consequently, individual *p*-values should be interpreted with caution. To minimize the risk of false positives, the analysis focused on features that showed consistent trends across both pathological groups and were supported by prior clinical or biological evidence. Future research involving larger and more diverse datasets will incorporate formal correction methods for multiple hypothesis testing to enhance statistical rigor.

While Haralick features are widely used in glaucoma and diabetic retinopathy detection studies, descriptive statistics (*μ* ± *σ*) of these features across different pathological groups are seldom reported. Most works focus on leveraging these features for classification tasks and do not provide quantitative comparisons between healthy and diseased groups (e.g., GLCM-based descriptors are listed but not summarized statistically) [12,34,35,36,37,38]. Reporting these values in the present study thus represents a novel contribution, and it underlines the need for future publications in this field to include group-wise feature distributions to facilitate comparative analysis and validation across datasets.

### 3.2. RGB Histogram and Color Distribution Analysis

**Figure 3:**
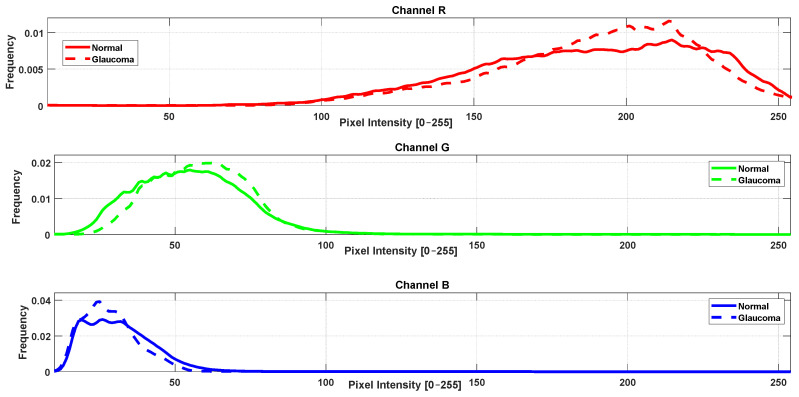
Average intensity histograms of red (R), green (G), and blue (B) channels for healthy and glaucoma groups.

**Figure 4:**
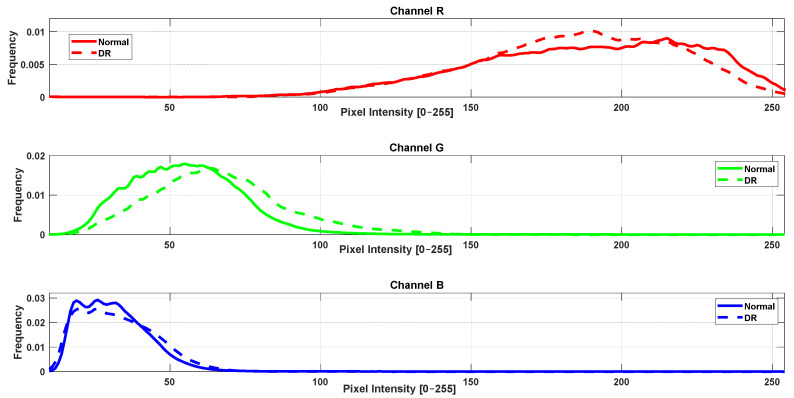
Average intensity histograms of red (R), green (G), and blue (B) channels for healthy and DR groups.

In parallel with the vascular texture analysis, a complementary study was conducted to investigate color distribution differences between healthy and pathological retinae using RGB histograms extracted from retinal fundus images of the same simples. For each image, the intensity histograms of the red (R), green (G), and blue (B) channels were computed individually. Subsequently, average histograms were calculated for each group (control, DR, glaucoma) and channel. Statistical comparisons were performed using MATLAB’s ranksum function (Wilcoxon rank-sum test). The results are illustrated in Figure 3 and Figure 4.

The analysis revealed statistically significant differences (*p* < 0.05) between the control group and DR group across all three RGB channels. When comparing the control group with the glaucoma group, significant differences were found in the green and blue channels. However, no significant difference was observed in the red channel between these two groups (*p* ≥ 0.05).

To evaluate multivariate color distribution differences between the control and pathological groups, the Mahalanobis distance was computed independently for each color channel. This analysis aimed to determine which RGB components most effectively differentiate healthy retinas from diseased ones.

The analysis follows these steps:

- Channelwise computation: Each color channel (R, G, B) was analyzed separately. Mahalanobis distance was calculated based on the histogram data for that specific channel only.
- Distributional comparison: For each channel, the Mahalanobis distance quantified how distinct the color distributions of the control group were from those of each pathological group, accounting for both mean and covariance structures.

The results are summarized in Table 3. A higher Mahalanobis distance indicates greater statistical separation between healthy and pathological distributions within a given channel. When combining all three channels, the overall Mahalanobis distance provides an integrated measure of color texture alteration, potentially reflecting structural or vascular changes associated with retinal pathology.

**Figure 5:**
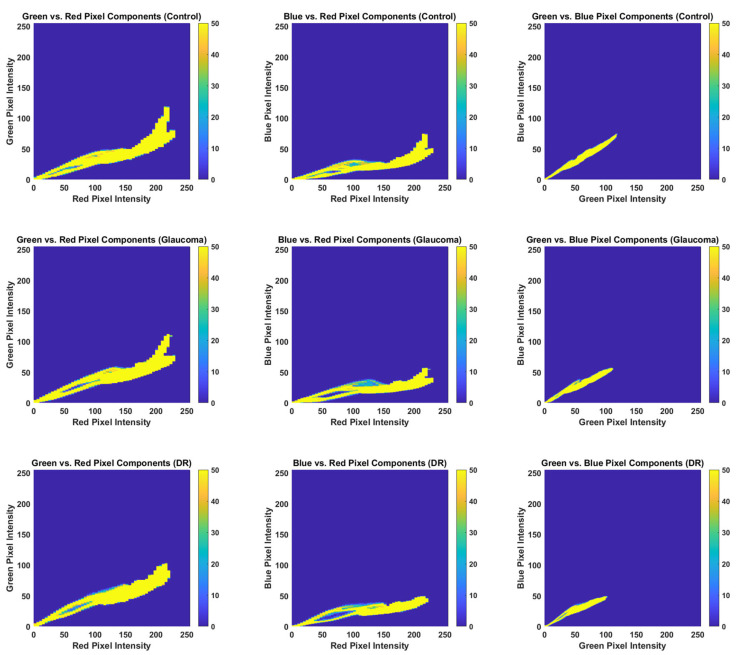
Bivariate histograms of the red, green, and blue RGB values for each pixel to visualize the color distribution.

To further explore color distribution patterns, bivariate histograms were generated for each image and are shown in Figure 5. These 2D histograms visualize the joint distribution of pixel values between pairs of color channels: red-green (RG), red-blue (RB), and green-blue (GB). Each plot includes a color scale, where yellow represents high-frequency regions and blue indicates low-frequency regions.

Across all groups, red remains the dominant color. However, the glaucoma and DR groups display clear shifts in the green and blue channel combinations compared to the control group, as evidenced by the broader and more diffuse yellow regions in the pathological histograms. These differences suggest changes in color composition likely to be tied to vascular or structural alterations.

Previous studies have demonstrated that color information is a valuable feature in the detection of various diseases [13,14]. The bivariate histogram analysis performed in this study supports the hypothesis that color distribution shifts, particularly in the green and blue channels, may serve as indicators of retinal disease, including glaucoma and DR.

This study demonstrates that combining Haralick texture features extracted from segmented retinal vasculature with global fundus color distribution analysis using RGB and bivariate histograms enables effective differentiation between healthy and pathological retinae affected by DR and glaucoma. The integration of these complementary structural and chromatic biomarkers provides a more comprehensive characterization of retinal abnormalities than approaches that analyze these features separately.

The novelty of this work lies in the dual-framework methodology applied to the same dataset, representing the first study to jointly leverage vessel-specific texture analysis and global color histogram modelling for retinal pathology assessment. Moreover, the use of multivariate statistical metrics, such as the Mahalanobis distance, to quantify group separability adds robustness and clinical relevance to the findings. This integrated approach advances the field by offering a non-invasive, image-based tool with potential for early detection, differentiation, and monitoring of retinal diseases in clinical and screening settings.

In summary, this study provides a statistical evaluation of texture and color features to assess their effectiveness in distinguishing between healthy and pathological retinae. While no machine learning methods were applied at this stage, the findings offer a solid foundation for future research involving supervised learning approaches to further validate the discriminative power of these features. Future work should also consider expanding the dataset to include a larger and more diverse population, as well as exploring the integration of complementary imaging modalities to enhance diagnostic performance.

## 4. Conclusions

This study demonstrated that quantitative texture analysis of the retinal vasculature, combined with global color distribution analysis of the fundus, enables effective discrimination between healthy and pathological retinae affected by DR and glaucoma. Haralick texture features extracted from segmented vascular structures captured relevant differences in structural organization. Pathological retinae showed significantly altered texture profiles, with increased energy and decreased entropy, correlation, and variance, indicators of reduced vascular complexity and tissue heterogeneity.

Simultaneously, RGB and bivariate histogram analyses of the full fundus area revealed consistent differences in color distribution between control and pathological groups. The green and blue channels showed statistically significant variation, and the Mahalanobis distance analysis confirmed that these color-based differences can be quantitatively measured.

The integration of these two approaches, vascular texture and fundus color analysis, offers a comprehensive, non-invasive method for characterizing retinal abnormalities. Notably, this is the first study, to the author’s knowledge, to apply both Haralick texture features and color histogram modelling to the same set of retinal images for disease classification purposes.

These findings support the potential of combining structural and chromatic biomarkers in retinal fundus imaging to aid in the early detection, differentiation, and monitoring of retinal pathologies.

While the sample size is limited, this study was conceived as a proof-of-concept to demonstrate the feasibility and diagnostic potential of combining vessel-specific texture features with global fundus color distribution metrics for retinal disease classification. The results underscore the strength of this dual-framework approach in capturing structural and chromatic alterations associated with retinal pathology.

Using 45 images from the HRF database allowed for a controlled and well-annotated setting but naturally restricts generalizability. To minimize bias, the dataset was balanced across groups and uniformly preprocessed.

Future work will focus on validating and extending this methodology with larger, more diverse datasets to assess its robustness, scalability, and clinical relevance in broader diagnostic and screening contexts.

Clinical Implications:

The results support the use of non-invasive texture and color-based biomarkers in the early screening and differential diagnosis of retinal diseases. The dual analysis approach offers a potential pathway for automated decision support systems in ophthalmology, particularly in resource-limited settings where fundus imaging is more accessible than advanced imaging modalities.

Limitations:

- The sample size, though representative, may limit generalizability across broader populations.
- The study was based on 2D fundus imaging, which does not capture depth information or fine capillary detail.
- While recent advances in retinal image analysis have been dominated by deep learning approaches such as DR-VNet and OCE-Net, this study focuses on classical handcrafted features, specifically vascular texture and color histogram analysis, which offer greater interpretability and computational efficiency. Direct benchmarking against these deep learning models was beyond the current scope due to dataset size and resource constraints. Nevertheless, this method provides a complementary perspective that can be particularly valuable in settings with limited annotated data.

Future Directions:

- Integration with deep learning frameworks could enable automated feature extraction and classification at scale.
- Further validation of larger and more diverse datasets from multiple clinical centers is needed to confirm robustness and reproducibility.

## Figures and Tables

**Table 1:** Haralick texture features calculated from GLCMs.

Texture Feature	Equation	
Energy or angular second moment	$\sum_{i=1}^{N}\sum_{j=1}^{N}P{\left(i,j\right)}^{2}$	(3)
Contrast	$\sum_{i=1}^{N}\sum_{j=1}^{N}{\left(i-j\right)}^{2}P(i,j)$	(4)
Correlation	$\sum_{i=1}^{N}\sum_{j=1}^{N}\left(\frac{i-\mu_{x}}{\sigma_{x}}\right)\left(\frac{j-\mu_{y}}{\sigma_{y}}\right)P(i,j)$	(5)
Variance	$\sum_{i=1}^{N}\sum_{j=1}^{N}{\left(i-\mu \right)}^{2}P(i,j)$	(6)
Sum Average	$\sum_{k=2}^{2N}kP_{x+y}(k);\begin{array}{cc} where & P_{x+y}(k)=\sum_{i=1}^{N}\underset{i+j=k}{\sum_{j=1}^{N}P(i,j)} \end{array}$	(7)
Sum variance	$\sum_{k=2}^{2N}{\left(k-\mu_{x+y}\right)}^{2}P_{x+y}(k);\begin{array}{cc} where & \mu_{x+y}=\sum_{k=2}^{2N}k.P_{x+y}(k) \end{array}$	(8)
Sum entropy	$-\sum_{k=2}^{2N}P_{x+y}(k)\log P_{x+y}(k)$	(9)
Entropy	$-\sum_{i=1}^{N}\sum_{j=1}^{N}P(i,j)\log P(i,j)$	(10)

**Table 2:** Results of Haralick texture features using co-occurrence matrix (comparison between healthy group and glaucomatous and DR groups).

Texture Feature	Healthy (μ±σ)	Glaucoma (μ±σ)	DR (μ±σ)	Diseased vs. Normal Controls (*p*-Value)
Energy	0.82 ± 0.02	0.86 ± 0.01	0.86 ± 0.02	<0.05
Contrast	0.52 ± 0.05	0.55 ± 0.06	0.52 ± 0.07	>0.05
Correlation	0.937 ± 0.005	0.912 ± 0.006	0.917 ± 0.006	<0.05
Variance	6.8 ± 0.6	5.3 ± 0.4	5.3 ± 0.6	<0.05
Sum Average	3.3 ± 0.1	2.96 ± 0.08	3.0 ± 0.1	<0.05
Sum variance	27.7 ± 2.0	19.1 ± 1.3	19.3 ± 2.2	<0.05
Sum entropy	0.36 ± 0.02	0.30 ± 0.02	0.30 ± 0.03	<0.05
Entropy	0.52 ± 0.03	0.44 ± 0.03	0.44 ± 0.05	<0.05

**Table 3:** Mahalanobis distance for each color channel. (R: red. G: green. B: blue).

	Mahalanobis Distance Per Channel		
	R	G	B
Healthy vs. glaucoma	3.45	3.50	4.20
Healthy vs. DR	4.26	3.88	3.56

## Data Availability

The original contributions presented in this study are included in the article. Further inquiries can be directed to the corresponding author.
